# Iron Oxide Nanoparticles: Multiwall Carbon Nanotube Composite Materials for Batch or Chromatographic Biomolecule Separation

**DOI:** 10.1186/s11671-021-03491-5

**Published:** 2021-02-10

**Authors:** Sebastian P. Schwaminger, Markus W. Brammen, Florian Zunhammer, Nicklas Däumler, Paula Fraga-García, Sonja Berensmeier

**Affiliations:** Bioseparation Engineering Group, Department of Mechanical Engineering, Technical University of Munich, Boltzmannstraße 15, 85748 Garching, Germany

**Keywords:** Iron oxides, Nanoparticles, Magnetic separation, Multiwall carbon nanotubes, Mixed-mode chromatography, Amino acids

## Abstract

Carbon-based materials are the spearhead of research in multiple fields of nanotechnology. Moreover, their role as stationary phase in chromatography is gaining relevance. We investigate a material consisting of multiwall carbon nanotubes (CNTs) and superparamagnetic iron oxide nanoparticles towards its use as a mixed-mode chromatography material. The idea is to immobilize the ion exchange material iron oxide on CNTs as a stable matrix for chromatography processes without a significant pressure drop. Iron oxide nanoparticles are synthesized and used to decorate the CNTs via a co-precipitation route. They bind to the walls of oxidized CNTs, thereby enabling to magnetically separate the composite material. This hybrid material is investigated with transmission electron microscopy, magnetometry, X-ray diffraction, X-ray photoelectron and Raman spectroscopy. Moreover, we determine its specific surface area and its wetting behavior. We also demonstrate its applicability as chromatography material for amino acid retention, describing the adsorption and desorption of different amino acids in a complex porous system surrounded by aqueous media. Thus, this material can be used as chromatographic matrix and as a magnetic batch adsorbent material due to the iron oxide nanoparticles. Our work contributes to current research on composite materials. Such materials are necessary for developing novel industrial applications or improving the performance of established processes.

## Introduction

Since the first synthesis of carbon nanotubes (CNTs) 1991 by Iijima [1] a matchless rise to one of the most powerful nanomaterials has begun. The production of CNT is based on the rolling of graphene layers to tubes. The electrical and mechanical properties of the CNTs can easily be tuned by different conformation of layers or multilayer assemblies. High elasticity, stability, thermal and electrical conductivity combined with a high specific surface area position CNTs to the spearhead of nanotechnology [2]. The applications of CNTs range from battery, sensors and high performance materials to drug delivery and wastewater treatment [2–9]. Many applications are based on the unique sorption properties of CNTs which possess a high specific surface area and a defined chemical structure. Long and Yang observed a strong adsorption behavior of the gases dioxane and nitrogen oxide while sulfur oxide bound moderately and carbon dioxide poorly to CNTs [10]. The highly hydrophobic surface of CNTs enables the possibility of binding nonpolar polymers or cyclic hydrocarbons via van-der-Waals interactions. Thus, CNTs can be employed as hydrophobic interaction chromatography (HIC) resin which was demonstrated by Biesaga and Pyrzynska who were able to purify dicamba herbicides by the use of CNTs as chromatography resin [11]. For electrochemically modulated chromatography applications and the control of ion exchange by a potential switch, CNTs represent a very promising stationary phase material [12, 13]. Furthermore, an application as extraction material for nonpolar compounds has been demonstrated [14]. However, the agglomeration of the CNTs can reduce the efficiency of extraction processes [15, 16]. Superparamagnetic iron oxide nanoparticles (SPIONs) also possess interesting adsorbent properties, as several applications in wastewater treatment or medicine demonstrate [17–19]. In wastewater treatment, iron oxide nanoparticles are used for e.g. heavy metal removal, due to their low cost, their high specific surface area and their complexation properties [20]. In medicine, iron oxides can be used as contrast agents for magnetic resonance imaging, as drug delivery agent or for hyperthermia applications [19, 21]. Hence, it is quite interesting to mix these two materials to combine their beneficial properties; especially the conductivity for CNTs and the superparamagnetism for SPIONs are valuable for numerous application fields [22]. The applications include e.g. magnetic solid phase extractions of dyes and pharmaceuticals [23–26]. The fundamental requirements of a large specific surface area are similar for solid phase extraction and liquid chromatography. Therefore, we want to test this material towards its suitability for chromatographic separation processes. Decorating CNTs with SPIONs is a possibility to enhance the dispersibility and the recyclability of CNTs due to the amphiphilic character of the surface [27]. Thus, a composite unites the mixed-mode functionality of CNTs and iron oxides. Additionally, the decoration affects magnetic and electric properties of this material [17, 18, 28–32]. Furthermore, due to the superparamagnetism of the SPIONs, magnetic separation is possible with the composite [33]. Ajayan and Iijima started mixing SPIONs and CNTs by filling the tubes with nanoparticles [34]. Other possibilities include the binding of SPIONs to the CNT via polymer linkers or emulsifiers [20, 35, 36]. The decoration is also possible by solvothermal synthesis of iron oxide nanoparticles and direct attachment to multiwall CNTs [37, 38]. Nowadays, the prevalent method for decorating CNTs with SPIONs is the here also used acidic carboxylation of CNTs before the SPIONs are synthesized or applied to coat the CNTs [39, 40]. Typically, the iron oxide nanoparticles are seeded by the carboxylized CNTs and can be co-precipitated or synthesized by the Fenton’s reaction directly on the surface [32, 41, 42]. While the carboxylation provides contact points for the SPIONs on the carbon surface, acidic treatment can lead to truncation of nanotubes [43]. Since the first combinations of SPIONs and CNTs multiple applications have been tested. The most challenging aspect in the synthesis is to control the aggregation of iron oxide nanoparticles in order to generate a homogeneous composite material [36, 43, 44]. Using carboxy groups as natural ligands for the iron oxide seeds for a co-precipitation process seems to be the best way to prevent strong aggregation effects and create a vastly decorated nanotube based material [32]. Therefore, we used established iron oxide synthesis routes for the attachment and decoration of carbon nanotubes [45, 46]. We investigated the surface modification of CNT with different acid treatments in order to improve the binding of iron oxide nanoparticles to the CNTs. Magnetic nanoparticles are mixed with the modified CNTs and the resulting materials are thoroughly characterized. Previous studies demonstrated the suitability of such materials as extraction matrix for the separation of organic compounds [47]. We investigate the use of the resulting material as chromatography resin and study the binding behavior of amino acids as analytes. This work highlights the relationship of the wetting behavior of synthesized materials with the chromatography results where the materials are used as stationary phase. Furthermore, our study emphasizes the use of chromatography to describe the surface properties of materials and offers a direction to exploit chromatography as a methodology for material characterization and understanding of interaction behavior in the future.

## Experimental

### Materials

Carbon nanotubes (Baytubes C 150 P) were obtained from Bayer Materials Science AG, Germany. Ferric chloride (FeCl_3_·6H_2_O) and sodium hydroxide (NaOH) were purchased from AppliChem GmbH, Germany. Ferrous chloride (FeCl_2_·4H_2_O) was purchased from Bernd Kraft GmbH, Germany. Hydrochloric acid, nitric acid, hydrogen peroxide and sulfuric acid were obtained from Sigma-Aldrich. All materials were used as obtained.

### Preparation of Carboxylated CNTs (cCNTs)

CNTs (10 g) were suspended in a mixture of concentrated nitric (67%) and sulfuric acid (98%) (1:3 v/v) and stirred at room temperature for 18 h. The product was diluted with deionized water to a total volume of 2 L in order to stop the reaction. The carboxylated CNTs were separated from the liquid with a paper filter and washed until a pH of 7 was reached. The resulting cCNTs were dried at 60 °C over night.

### Co-precipitation of SPIONs on cCNTs

For the decoration of cCNT with MNP, a similar approach as described by Baykal et al. 2013 was chosen [32]. Dried cCNTs (2 g) were ultrasonicated in 800 mL deionized water in order to disagglomerate the tubes. The suspension was held at room temperature, stirred at 350 rpm and mixed with 14 g FeCl_3_ 6 H_2_O and 5.2 g FeCl_2_ 4H_2_O. Sodium hydroxide (2 mol L^−1^) was added to the suspension until a pH of 9.5 had been reached. The reaction was stopped after 30 min and the solid was filtered. The SPION decorated cCNTs are lyophilized in a freeze-drier Alpha 1–2 LDplus (Martin Christ Gefriertrocknungsmaschinen GmbH) prior to further analysis.

### Methods

#### Transmission Electron Microscopy (TEM)

Low amounts of dried nanotubes were suspended in deionized water and disagglomerated with a Branson sonifier. The suspension was precipitated on a TEM grid and analyzed with a JEOL 100 CX. The micrographs were analyzed and a minimum of 100 particles were counted on each picture.

#### X-Ray Diffraction (XRD)

Dried samples were measured by a Stadi P diffractometer (STOE & Cie GmbH, Germany) equipped with a MoKα (*λ* = 0.7093 Å) source in transmission geometry. Data was collected in the range from 2° to 50° (2ϴ). The software package STOE WinXPOW (STOE & Cie GmbH, Germany) was used for indexing and refinement purposes. The full width at half maximum and the position of the ⟨2 2 0⟩ reflections were used to determine the primary particle diameter according to the Scherrer equation. A factor of 0.89, which is in agreement with spherical particles, was chosen.

#### X-Ray Photoelectron Microscopy (XPS)

X-ray photoelectron spectroscopy was accomplished with a Leybold–Heraeus LHS 10 XPS system in ultrahigh vacuum (UHV) hosting a non-monochromatized Al Kα source (1486.7 eV). The powder samples were fixed on a vacuum compatible copper foil adhesive tape. The spectra were recorded at a constant pass energy mode set to 100 eV and a full width at half-maximum (FWHM) of ~ 1.1 eV. The C 1*s* (284.5 eV) peak corresponding to adventitious carbon was used as energy spectra of the C 1*s*; O 1*s* and Fe 2*p* regions were acquired by repeatedly scanning the same region 30 times in order to reduce statistical noise. All spectra were recorded in a UHV at a pressure below 5 × 10^−8^ mbar. The core level spectra were fitted by a mix of Gaussian and Lorentzian functions (Gaussian line width (0.7 eV) and Lorentzian line width (0.3 eV)).

#### Tensiometry

The contact angles of CNTs, cCNTs and cCNT-SPIONs were measured with a Krüss T100 MK3 tensiometer. Therefore, a packed bed of the nanotubes with a height of 2 cm was compressed uniformly for all samples. The capillarity of the packed beds was determined with the dissemination of n-hexane. Contact angles were determined with the liquids diiodo methane, dimethylsulfoxide, ethylene glycole, glycerine and deionized water. The free surface energy was calculated with the OWRK (Owens Wendt Rabel and Kälble) method [48].

#### Adsorption Experiments

Adsorption isotherms of l-lysine at different concentrations in 100 mM phosphate buffer at pH 7.8 were conducted with SPIONs, cCNTs and cCNT-SPIONs. The amino acid was incubated for 24 h with the adsorbent and vigorously shaken at 25 °C. Different concentrations of the amino acid were incubated with 1 g L^−1^ of cCNTs or cCNT-SPIONs and with 2 g L^−1^ SPIONs. The supernatant concentration was determined by an assay based on the Cayot method. This method is based on the photometric detection at 420 nm after modification of an amino acid with TNBSA at pH 8.5 [49].

#### Chromatography Experiments

The dynamic binding capacities (DBC) of CNTs, cCNTs and cCNT-SPIONs were determined with a chromatography column (Omnifit) with a diameter of 6.6 mm which is adjustable from both sides and equipped with a 25 µm PE frit. All samples were packed dynamically under flowing water until a height of 6 to 8 cm is reached. The chromatography experiments were conducted at a flow of 0.3 mL min^−1^. The height equivalent to a theoretical plate (HETP) and the dead time were determined with a 1 M NaCl solution according to a modified van Deemter Equation: $\text{HETP} = {{L}}\frac{{ \sigma }^{2}}{{\mu}^{2}}$; *L* is the column length, *σ* represents the variance of the chromatography peak and *μ* is the first peak moment. Furthermore, the asymmetry of the packed column was evaluated at 10% peak height prior to conducting the experiments: ${{A}}_{{s}}=\frac{{b}}{{{a}}}$; *a* represents the width of the front part of the peak divided at peak maximum and *b* the width of the rear part. For calculating the DBCs of different amino acids (glycine, l-lysine, l-histidine, l-glutamic acid and l-cysteine), the solutions were adjusted to 10 mM at pH 6 with HCl or NaOH. The amino acids were detected with a diode array detector at 200 nm. The columns were washed with 30 mL water prior to loading with 15 mL of amino acid solution and washing with 20 mL of water followed by an elution with 20 mL of 1 M NaCl and another regeneration step of 20 mL water. The dynamic binding capacity was measured at 10% of the peak maximum. All experiments have been conducted in triplicates.

## Results and Discussion

Multiwall carbon nanotubes are quite hydrophobic and therefore tend not to interact strongly with polar iron oxide nanoparticles. In order to make the CNTs more polar and allow for interactions with iron oxides, the surface was treated with nitric and sulfuric acid. This treatment generates surface defects and even charged surface groups, which act as seeds nanoparticle co-precipitation and as binding sites for iron oxide nanoparticles. Several methods and agents for the generation of defect sites were tested and analyzed with Raman spectroscopy (Additional file 1: Fig. S1). Raman spectroscopy yields the ratios of the defect band (*D*) to the graphite band (*G*) [41, 42, 50, 51]. The ratio of the integral of *G* to *D* band is dependent on the laser wave length used [52]. However, the increase in this ratio is usually a good indicator for the oxidation of the nanotube surface and thus to identifying the best method for surface modification of CNTs [52–55]. While our results indicate a very high ratio for *D*:*G* after nitric acid treatment of the nanotubes suspension, the mix of nitric and sulfuric acid lead to a slight increase of this ratio (Additional file 1: Table S1). For the further experiments only CNTs treated with the mix of nitric and sulfuric acid were used which represented the most reproducible method leading to nanotubes which can be packed in chromatography columns in accordance to our experiments and literature [54]. These nanotubes are referred to as carboxylated CNTs (cCNTs).

**Fig. 1:**
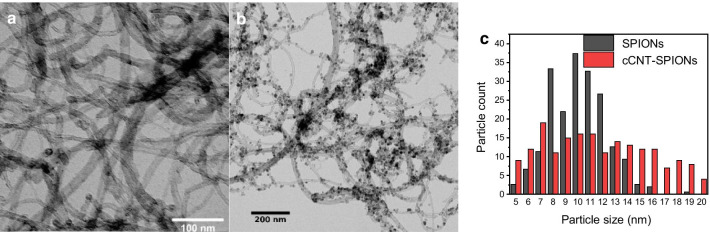
TEM images of cCNTs (**a**) and cCNT-SPIONs (**b**). Size distribution from four images and a count of minimum 30 particles per picture for each material **c**

Iron oxide nanoparticles synthesized by co-precipitation in presence of cCNTs yield SPIONs with slightly larger average size as similarly synthesized particles without cCNTs (Fig. 1c). However, the size distribution is broader in the range of 5–20 nm and the particles synthesized with cCNTs are attached to the nanotubes. The synthesized composite material mainly possesses the properties of the iron oxide nanoparticles and is decorated homogeneously. The decoration of cCNTs with iron oxide nanoparticles, which are present as dark spots, can be observed with transmission electron microscopy is in good agreement with literature [32, 36–38]. Our results indicate a homogeneous load of SPIONs on cCNTs as no larger aggregates of nanoparticles can be observed in the images (Fig. 1b).

**Fig. 2:**
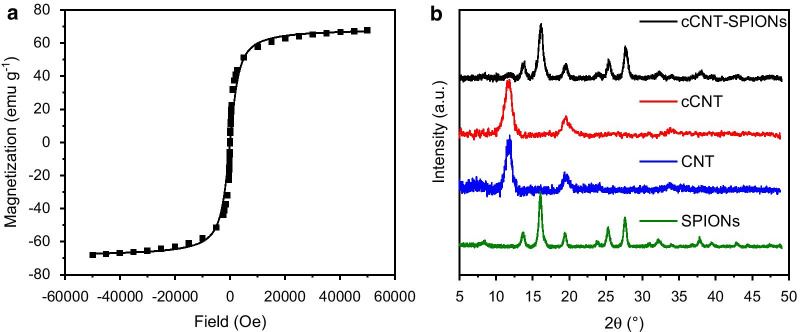
Magnetic hysteresis curve obtained with a SQUID from -50,000 to 50,000 Oe at 300 K (**a**) and powder XRD patterns of cCNT-SPIONs, cCNTs, CNTs and SPIONs obtained with a MoKα source (**b**)

Magnetization measurements at room temperature indicate a superparamagnetic composite material with a saturation magnetization of 67 emu g^−1^ and no magnetic remanence (< 1 emu g^−1^). The saturation magnetization is only slightly lower than pure bare iron oxide nanoparticles and the shape of the hysteresis curve is similar (Fig. 2) [45, 56, 57]. Hence, surface layer and core composition of SPIONs and cCNT-SPIONs composites are similar [57]. The crystallographic analysis of the composite material shows a spinel structure corresponding to either magnetite or maghemite, while no crystalline structure of CNTs can be observed (Fig. 2b) [37, 57]. The signal at 11.8° occurring in CNTs and cCNTs can be indexed as the ⟨0 0 2⟩ reflection of the hexagonal graphite structure [17]. The decoration of cCNTs with iron oxide nanoparticles leads to the occurrence of the reflections:  ⟨1 1 1⟩ at 5.4° ⟨2 2 0⟩ at 13.7°, ⟨3 1 1⟩ at 16.1°, ⟨4 0 0⟩ at 19.4°, ⟨4 2 2⟩ at 23.8°, ⟨5 1 1⟩ at 25.3° and ⟨4 4 0⟩ at 25.6° [29, 41]. These reflections are consistent with the standard XRD data for the cubic phase Fe_3_O_4_ (JCPDS no. 89-4319) with a face-centered cubic structure and our own reference SPIONs [17, 32]. The reflections of the composite material show a larger FWHM resulting in a smaller Scherrer diameter of the crystalline material. This behavior can be explained with the larger particle size distribution of the composite material as observed with TEM, and the additional nucleation seeds on the surface of cCNTs [46]. Higher numbers of nucleation seeds usually lead to smaller primary crystallites [36, 46]. The magnetization as well as the diffraction patterns are in good agreement with other decoration methods such as electrostatic modification through polyethyleneimine or polyacrylate acid [18, 22]. The intensity of the reflection corresponding to graphite is similar in the pure material and the composite material and no significant changes can be observed. However, the intensity of the reflections corresponding to iron oxide structures demonstrate a much more intense signal.

**Fig. 3:**
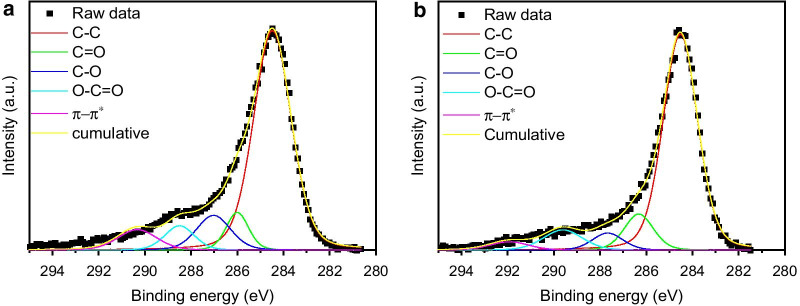
XP spectra in the range of C 1*s* of cCNTs (**a**) and cCNT-SPIONs (**b**). The spectra are fitted with a combination of Gaussian and Lorentzian functions with the program Origin

Hence, the composite material combines the properties of carbon nanotubes and magnetic nanoparticles. X-ray photoelectron spectroscopy, which is a very surface sensitive method shows the attachment of iron oxide to cCNTs (Additional file 1: Fig. S2). Here, magnetite and/or maghemite are present since the F 2*p*^3/2^ band of the cCNT-SPIONs shows a maximum at 711 eV and the Fe 2*p*^1/2^ shows a maximum at 724 eV [37, 57]. The shape of the shake up satellites also indicates the presence of magnetite rather than hematite [57, 58]. The O 1*s* region indicates C–O, and COO^−^ bonds for both cCNTs and cCNT-SPIONs, while the composite material demonstrates another band at 529.5 eV which corresponds to the presence of Fe–O bonds [37, 41]. The bonds between carbon and oxygen in the O 1s region are in good agreement with the observations of the C 1*s* region, which also indicates differently oxidized carbon species (Fig. 3). Here, not only carboxy groups (289 eV) but other C–O bonds (286–287.5 eV) as well as *sp*^2^ hybridized carbon (284.5 eV) corresponding to carbon from the backbone structure of CNTs can be observed [41, 51].

**Fig. 4:**
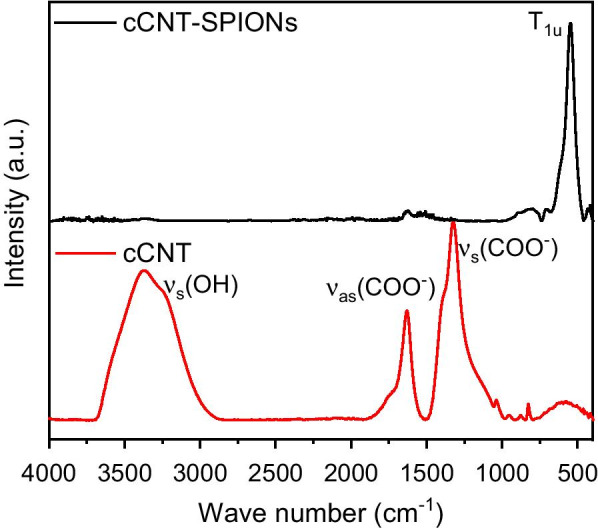
ATR-IR spectra of cCNT-SPIONs and cCNTs

The decoration of cCNTs with SPIONS can be observed with ATR-IR spectroscopy as well. In Fig. 4, the band at 550 cm^−1^ corresponding to a T_1u_ vibration of magnetite crystals is most prominent for the composite material [17, 58]. On the carboxylized CNTs the COO^−^ symmetric and asymmetric stretch vibrations corresponding to the carboxy group can be observed at 1325, 1400 (s) and 1624 cm^−1^ (as), respectively [17, 36, 50, 59]. Furthermore, the O–H stretch vibrations around 3250 cm^−1^ indicate the presence of carboxy groups on the cCNTs [17, 29, 32, 50]. The decrease of intensity for the peaks corresponding to C–O vibrations for cCNT-SPIONs combined with the prominent band corresponding to Fe–O vibrations is a good indicator for a homogeneous coating. Iron oxide nanoparticles are the better absorber of infrared radiation and therefore the results are not quantitative but only an indicator of an increase in iron oxide on the surface combined with a disappearance of bands corresponding to C–O vibrations.

**Fig. 5:**
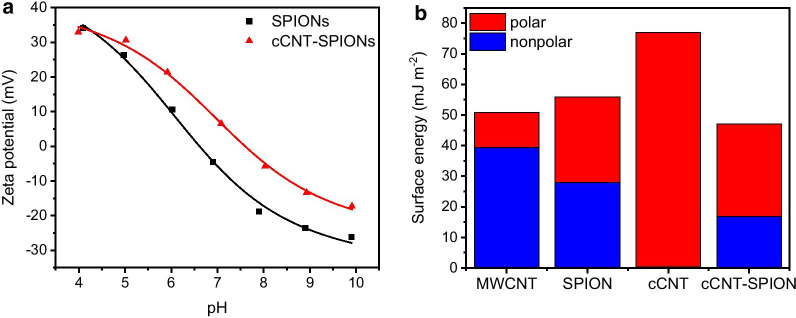
Zeta potential of SPIONs and cCNT-SPIONs from pH 4 to 10 (**a**) and surface free energy obtained from capillary rise experiments with the OWRK method (**b**)

The zeta potential of the composite material is only slightly higher than the zeta potential of bare SPIONs, even though the acidic treatment should result in a highly negatively charged material. We were not able to conduct zeta potential measurements of CNTs which tend to aggregate in aqueous environments. The isoelectric point of the composite material is still in the neutral range with pH 7.5 compared to pH 6.5 for the bare nanoparticles (Fig. 5a) [57]. This behavior suggests a good coating or decoration of the cCNTs with iron oxide nanoparticles and supports the other analytical characterizations of the composite. The occurrence of iron in the XP spectra, the appearance of Fe–O vibrations in the IR spectrum, the high saturation magnetization and the occurrence of higher density materials in TEM all point to a successful attachment of iron oxide nanoparticles to cCNTs. Furthermore, the behavior of the zeta potential is amphiphilic and similar for both materials with increasing and decreasing pH, which also indicates iron oxides being the most prominent surface species. We observe a very similar behavior for wetting experiments with the capillary rise method (Additional file 1: Fig. S3). Here, the surface free energy as well as the polar and dispersive share, yielded by multiple experiments with different solvents, are similar for composite and bare iron oxide nanoparticles. The capillarity of the materials is determined with ethylene glycole for bare carbon nanotubes as described in literature [60, 61]. For all other materials, hexane as fluid with a very low surface tension (18.4 mJ m^−2^) was chosen to determine the capillarity. Bare iron oxide nanoparticles possess a total surface free energy of 55.9 mJ m^−2^, while the composite material has a surface energy of 47.1 mJ m^−2^ (Fig. 5b). The composite material has a slightly higher polar share, However, the untreated CNTs and the cCNT behave completely different. The untreated CNTs show a high dispersive surface free energy, while the cCNTs are highly polar according to the method from Owens Wendt Rabel and Kälble [48]. From this method the polar and dispersive shares of the wetting liquids and the resulting contact angle derived from capillary rise experiments can be compared (Additional file 1: Fig. S4). The surface free energy of the CNTs obtained from this method is slightly higher than the results obtained from Dresel and Teipel who also performed capillary rise experiments with Baytube CNTs [60]. The tensiometry results obtained with the capillary rise method are an interesting indicator in order to describe the differences such as polarity and wetting with water of nanomaterial surfaces. However, especially with nanostructured surfaces and nanostructured capillaries, this method can be prone to errors. Here, all materials show a high specific surface area (Additional file 1: Figure S5). Bare iron oxide nanoparticles demonstrate a specific surface area of 110 m^2^ g^−1^, the cCNTs possess a specific surface area of 228 m^2^ g^−1^ and the composite material shows a specific surface area of 131 m^2^ g^−1^. This specific surface area is in a similar range, especially when considering the volumetric surface area since the densities of cCNTs (1.46 g cm^−1^), cCNT-SPIONs (2.38 g cm^−1^) and SPIONs (3.8 g cm^−1^) vary significantly. The density of CNTs is in good agreement within the variation of density for carbon nanotubes [53].

**Fig. 6:**
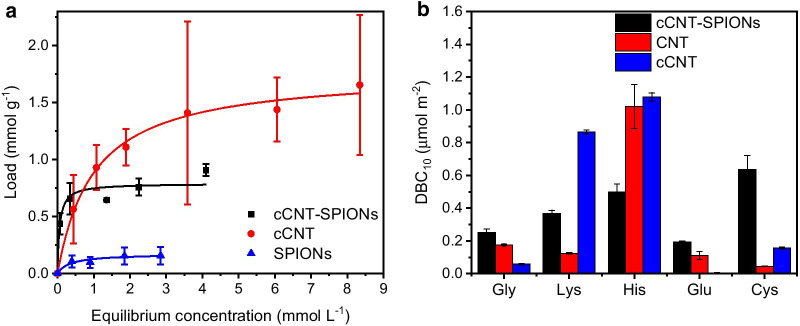
Static binding capacities of l-lysine with cCNTs, cCNT-SPIONs and SPIONs at pH 7.8 with 100 mM phosphate buffer (**a**). Dynamic binding capacities at 10% of the breakthrough of cCNT-SPIONs, CNTs and cCNTs obtained from inverse chromatography experiments with different amino acids at pH 6 (**b**)

In order to make use of the composite material for solid phase extraction and chromatography processes, the static and dynamic binding capacity of amino acids has been compared. One of the main goals of this study was to verify if the composite material can be used in a chromatography system, where iron oxide nanoparticles can act as stationary phase. The bare nanoparticles cannot be used as a stationary phase in a chromatography column on their own due to the large pressure drop and possible losses through the filter. Thus, the binding behavior of amino acids to bare nanoparticles can only be compared with adsorption isotherms in equilibrium. In Fig. 6a, a similar behavior of the adsorption isotherm of l-lysine on cCNT-SPIONs, and SPIONs can be observed. The equilibrium binding constant (*K*_*D*_) is in a similar range (0.17 g L^−1^ for cCNT-SPIONs and 0.72 g L^−1^ for SPIONs) and the large difference in the maximum binding capacity (0.91 g g^−1^ for cCNT-SPIONs and 0.15 g g^−1^ for SPIONs). The differences of affinity and binding capacity can be explained by strong electrostatic interactions between lysine and negatively charged materials such as cCNTs compared to the interactions with amphiphilic iron oxides [62]. However, iron oxide nanoparticles are also usually negatively charged as they get complexed by phosphate ions in PBS buffer [63]. l-lysine was chosen since this amino acid is much easier to detect with the TNBSA method compared to other amino acids [49]. The maximum load is in a similar range as literature for cationic adsorptives such as methylene blue or aniline on iron oxide decorated carbon nanotubes [17, 51]. For the dynamic binding capacity, which was obtained from inverse liquid chromatography experiments, huge differences between cCNTs and cCNT-SPIONs can be observed. While amino acids such as the positively charged l-lysine shows a higher DBC on cCNTs, the negatively charged l-glutamate shows a significantly higher DBC on cCNT-SPIONs. This behavior is in good agreement with literature, where l-glutamate demonstrates a high affinity to iron oxide nanoparticles [59, 64]. The high affinity of the l-lysine to cCNTs can be explained by electrostatic interactions between the positively charged amino acid and the negatively charged carboxy functionalized nanotubes. Glycine shows a higher dynamic binding capacity to cCNT-SPIONs than to cCNTs, which might be explained by the more amphiphilic character of iron oxide surfaces. The high dynamic binding capacity of l-cysteine to the composite material is in good agreement with the literature and the formation of cystine due to interaction of l-cysteine with iron ions [59]. l-histidine shows a high dynamic binding capacity to all materials since electrostatic, coordinative and hydrophobic interactions are possible. While the column packed with cCNT-SPIONs shows a higher HETP value compared to CNTs and cCNTs, the asymmetry of cCNT-SPIONs is similar to cCNTs and with around 0.7 in a range which allows the analysis of breakthrough curves (Additional file 1: Fig. S6 and Table S2). The porosity of all systems is in a similar range between 0.78 and 0.94 and in good agreement with other stationary phases used for inverse chromatography experiments [65].

## Conclusion

In this study, a composite material combining superparamagnetic iron oxide nanoparticles and carbon nanotubes has been synthesized. The first aim of this study was to investigate the surface properties of the composite material with the goal of understanding the share of each initial material to the final composite. Furthermore, the study is a proof of concept to test the effect of such materials for molecule separation with focus on the retention behavior of amino acids with liquid chromatography. The idea was to combine the surface reactivity of iron oxide nanoparticles with the packing properties of carbon nanotubes, a chromatographic matrix which leads to very low pressure drop. It was possible to establish a chromatography system and characterize the interaction of positively or negatively charged, and uncharged amino acids with the composite material. Hence, this material might be a good indicator for interactions with a CNT-basedmatrix. However, not only the use in a chromatography system but also processes such as solid phase extraction are possible with the created material due to the high saturation magnetization obtained with the described decoration procedure [33]. The magnetic properties allow for a simple magnetic separation, while the carbon nanotubes regulate the macroscopic structure and the accessibility of target molecules to the surface. With this study we want to emphasize the similarity of magnetic separation and analytical chromatography since similar materials and adsorption equilibria can be demonstrated, even though there are multiple differences. For the future exploitation of this unique magnetic material, particularly its hydrodynamic properties seem interesting and should be analyzed, e.g. for mixed-mode applications as in chromatography. Furthermore, the electrical properties of the composite might pave the way for further electrochemical applications. Tabassum et al. reviewed multiple applications for metal-based nanoparticles confined into carbon nanotubes, which open up opportunities for electro-catalysis, energy conversion and storage devices [66].

The understanding and design of composite materials and the description of surface and interface properties is challenging. Nevertheless, composite materials have the power to open doors for higher complexity in applications in all fields in the future. Chromatography is somehow a pioneering technology, which shows applicability for all possible kinds of target compounds and offers a very broad portfolio of methods and of processing solutions. We think that materials as the one we present in this study are necessary to understand the share of different properties in a particular processing form and how materials of different composition impact the final output of processes based on interactions at the solid–liquid interface.

## Supplementary Information

**Additional file 1.** **Fig. S1.** Whole recorded Raman spectra of CNTs oxidized by different agents. **Table S1.** Integrals of diamond (D), graphite (G) and Diamond' (D') bands. **Fig. S2.** XP spectra of cCNT and cCNT-SPIONS: Survey spectra a, spectrum of cCNT-SPIONs in the Fe 2p region b and spectra in the O 1s region of cCNT c and cCNT-SPIONS d. **Fig. S3.** Wetting of nanomaterials with different solvents: CNTs a cCNTs b cCNT-SPIONs c. **Fig. S4.** Plot for the calculation of the surface free energy in accordance to the OWRK (Owens-Wendt-Rabel-Kälble) method. **Fig. S5.** Adsorption isotherms of nitrogen on cCNTs, SPIONs, and cCNT-SPIONs at 77 K. **Fig. S6.** Voidage and porosity determination of packed columns with a NaCl tracer for cCNTs a, cCNT-SPIONs b and CNTs c. The volume flow is 0.3 mL min-1 for cCNTs, cCNT-SPIONs and 0.5 mL min-1 for CNTs leading to a dead time of 3.8 and 2.3 min, respectively. The concentration of the NaCl tracer is 0.5 and 5 M, respectively. The chromatograms are fitted with an exponentially modified Gaussian (EMG) fit. **Table S2.** Parameters of the chromatography column for the different packings.

## Data Availability

All data generated or analyzed during this study are included in this published article and its supplementary information files.

## Abbreviations

BET: Brunauer Emmet Teller
cCNT: Carboxylized carbon nanotubes
cCNT-SPIONs: Carboxylized carbon nanotubes and attached superparamagnetic iron oxide nanoparticles
CNTs: Carbon nanotubes
D band: Diamond band
DBC: Dynamic binding capacity
EMG: Exponentially modified Gauss
FWHM: Full width at half maximum
HETP: Height equivalent to a theoretical plate
G band: Graphite band
OWRK: Owens Wendt Rabel Kälble
PBS: Phosphate buffered saline
SPIONs: Superparamagnetic iron oxide nanoparticles
TEM: Transmission electron microscope
TNBSA: 2,4,6-Trinitrobenzene sulfonic acid
UHV: Ultra-high vacuum
XPS: X-ray photoelectron spectroscopy
XRD: X-ray diffraction
