# Supplementary material for: Iron Oxide Nanoparticles: Multiwall Carbon Nanotube Composite Materials for Batch or Chromatographic Biomolecule Separation
Source: Nanoscale Res Lett. 2021 Feb 10;16:30. doi: 10.1186/s11671-021-03491-5 (PMC7876204; doi:10.1186/s11671-021-03491-5)
Supplement: Supplementary file 1 — Additional file 1. Fig. S1. Whole recorded Raman spectra of CNTs oxidized by different agents. Table S1. Integrals of diamond (D), graphite (G) and Diamond' (D') bands. Fig. S2. XP spectra of cCNT and cCNT-SPIONS: Survey spectra a, spectrum of cCNT-SPIONs in the Fe 2p region b and spectra in the O 1s region of cCNT c and cCNT-SPIONS d. Fig. S3. Wetting of nanomaterials with different solvents: CNTs a cCNTs b cCNT-SPIONs c. Fig. S4. Plot for the calculation of the surface free energy in accordance to the OWRK (Owens-Wendt-Rabel-Kälble) method. Fig. S5. Adsorption isotherms of nitrogen on cCNTs, SPIONs, and cCNT-SPIONs at 77 K. Fig. S6. Voidage and porosity determination of packed columns with a NaCl tracer for cCNTs a, cCNT-SPIONs b and CNTs c. The volume flow is 0.3 mL min-1 for cCNTs, cCNT-SPIONs and 0.5 mL min-1 for CNTs leading to a dead time of 3.8 and 2.3 min, respectively. The concentration of the NaCl tracer is 0.5 and 5 M, respectively. The chromatograms are fitted with an exponentially modified Gaussian (EMG) fit. Table S2. Parameters of the chromatography column for the different packings. [file 11671_2021_3491_MOESM1_ESM.docx]

**Supplementary Material**

Iron oxide nanoparticle - multiwall carbon nanotube composite materials for batch or chromatographic biomolecule separation

Sebastian P. Schwaminger*, Markus W. Brammen, Florian Zunhammer, Nicklas Däumler, Paula Fraga-García, Sonja Berensmeier*

Bioseparation Engineering Group, Technische Universität München, Boltzmannstraße 15, Garching D-85748, Germany

*E-mail address: [s.berensmeier@tum.de](mailto:s.berensmeier@tum.de)

*E-mail address: [s.schwaminger@tum.de](mailto:s.schwaminger@tum.de)

**Raman spectroscopy**

Powder and suspension samples were measured with a SENTERRA Raman spectrometer (Bruker Optics GmbH, Germany). The samples were recorded at a power of 0.4 mW with a 488 nm laser source and an objective with a magnification of 50x.

**Determination of the specific surface area by BET-isotherms**

### For nitrogen adsorption isotherms, a Gemini VII 2390 Surface Area Analyzer (Micromeritics Instrument Corporation, USA) was used. Prior to analysis, the samples were dried in vacuum at 100 °C and weighted. The measurement included a determination of the sample volume with the inert gas helium and the gas adsorption isotherm of nitrogen at 77 K. From the gas adsorption isotherms the specific surface area was determined by the BET-method in a relative pressure range between 0.05 and 0.25 mbar.

**Determination of the density**

### In order to determine the density of nanomaterials, the dried powders were weighed with the Krüss K100 tensiometer on a special density measurement tool. The powders were then dived into ethanol to determine the displacement of solvent and thus determine the densities. This process yielded a density of 3.8 g cm^-1^ for bare iron oxide nanoparticles, 1.46 g cm^-1^ for cCNTs and 2.38 g cm^-1^ for the composite cCNT-SPIONs.

**Fig. S1** Whole recorded Raman spectra of CNTs oxidized by different agents.

**Table S1** Integrals of diamond (D), graphite (G) and Diamond' (D') bands.

|  | D band (integral) | G band (integral) | D' band (integral) | Ratio D/G |
| --- | --- | --- | --- | --- |
| CNT | 85.5 | 64.4 | 6 | 1.33 |
| HCl | 86.2 | 71.5 | 5.1 | 1.21 |
| HNO_3_ | 87.5 | 57.9 | 15.8 | 1.51 |
| H_2_SO_4_/HNO_3_ | 84.9 | 44.4 | 9.6 | 1.91 |
| NH_4_OH+H_2_O_2_ | 85.6 | 61.7 | 8.4 | 1.39 |

c

d

b

a

**Fig. S2** XP spectra of cCNT and cCNT-SPIONS: Survey spectra **a**, spectrum of cCNT-SPIONs in the Fe 2p region **b** and spectra in the O 1s region of cCNT **c** and cCNT-SPIONS **d**.

###

c

b

a

### Fig. S3 Wetting of nanomaterials with different solvents: CNTs a cCNTs b cCNT-SPIONs c.

###

### Fig. S4 Plot for the calculation of the surface free energy in accordance to the OWRK (Owens-Wendt-Rabel-Kälble) method.

###

### Fig. S5 Adsorption isotherms of nitrogen on cCNTs, SPIONs, and cCNT-SPIONs at 77 K.

###

b

c

a

### Fig. S6 Voidage and porosity determination of packed columns with a NaCl tracer for cCNTs a, cCNT-SPIONs b and CNTs c. The volume flow is 0.3 mL min^-1^ for cCNTs, cCNT-SPIONs and 0.5 mL min-1 for CNTs leading to a dead time of 3.8 and 2.3 min, respectively. The concentration of the NaCl tracer is 0.5 and 5 M, respectively. The chromatograms are fitted with an exponentially modified Gaussian (EMG) fit.

### Table S2 Parameters of the chromatography column for the different packings.

| Sample | Column length [cm] | HETP (H) [mm] | Plate count (N) | Asymmetry (A_s_) | Porosity (ε) |
| --- | --- | --- | --- | --- | --- |
| cCNTs | 6 | 2.59 | 23.18 | 0.61 | 0.78 |
| cCNT-SPIONs | 5 | 7.22 | 7 | 0.597 | 0.94 |
| CNTs | 6.5 | 2.90 | 22.42 | 1.64 | 0.90 |
